# Is takeout culture associated with rising colorectal cancer in young Chinese adults? An ecological analysis of GBD and CHNS data

**DOI:** 10.3389/fpubh.2025.1665377

**Published:** 2025-10-23

**Authors:** Ling Yao, Yufei Li, Yuqiao Yao, Guixiang Qian, Zhuhui Zhang, Qiuling Liu, Jie Dai, Xuedi Lei, Xiaoqiang Jia, Longfang Quan, Haixia Li, Yonghai Li

**Affiliations:** ^1^Department of Anorectal, Guang’anmen Hospital, China Academy of Chinese Medical Sciences, Beijing, China; ^2^Chizhou Second People’s Hospital, Hefei, Anhui, China; ^3^The First Affiliated Hospital of USTC, Division of Life Sciences and Medicine, University of Science and Technology of China, Hefei, Anhui, China; ^4^Department of Anorectal, Xi Yuan Hospital, China Academy of Chinese Medical Sciences, Beijing, China; ^5^Bengbu Medical University, Bengbu, Anhui, China; ^6^Department of Cardiology, Guang’anmen Hospital, China Academy of Chinese Medical Sciences, Beijing, China; ^7^Department of Anorectal, The First People’s Hospital of Hefei, Hefei, Anhui, China

**Keywords:** nutritional deficiency, gastrointestinal diseases, incidence, DALYs, dietary habits, GBD, CHNS

## Abstract

**Objective:**

To explore the epidemiology of diet-related diseases and shifts in dietary habits among frequent takeout consumers in China over the previous decade.

**Methods:**

This study utilized data from the Global Burden of Disease (GBD) 2021 to analyze incidence trends of 12 diet-related diseases, including nutritional deficiencies, non-neoplastic digestive diseases, and gastrointestinal cancers, spanning from 2010 to 2021. Additionally, we assessed changes in Disability-Adjusted Life Years (DALYs) attributable to various risk factors. Changes in dietary behavior among young Chinese adults were evaluated using data from the China Health and Nutrition Survey (CHNS).

**Results:**

The standardized incidence of Vitamin A deficiency has consistently declined over the past decade. Among non-neoplastic digestive diseases, inflammatory bowel disease showed the most pronounced decrease. In contrast, the burden of colorectal cancer has increased annually across all age groups. Significant risk factors for colorectal cancer—including low intake of whole grains and milk, and high consumption of red and processed meats, as well as smoking—have contributed to an increase in DALYs in 2021 compared to 2010. Additionally, these shifts in beverage consumption—particularly the steep rise in soft drinks—are a key public health concern given their established dietary risks. Critically, their temporal coincidence with the explosive growth of the takeout market posits the delivery ecosystem as a potential amplifier of these unhealthy dietary patterns.

**Conclusion:**

From 2010 to 2021, colorectal cancer incidence rose among Chinese adults aged 20–39, despite declining burdens of nutritional deficiencies and non-neoplastic digestive diseases. This trend parallels shifts in dietary behavior and the rapid growth of the takeout industry. These findings call for further research into causal links and platform-based public health interventions promoting healthier diets.

## Graphical abstract

**Figure:**
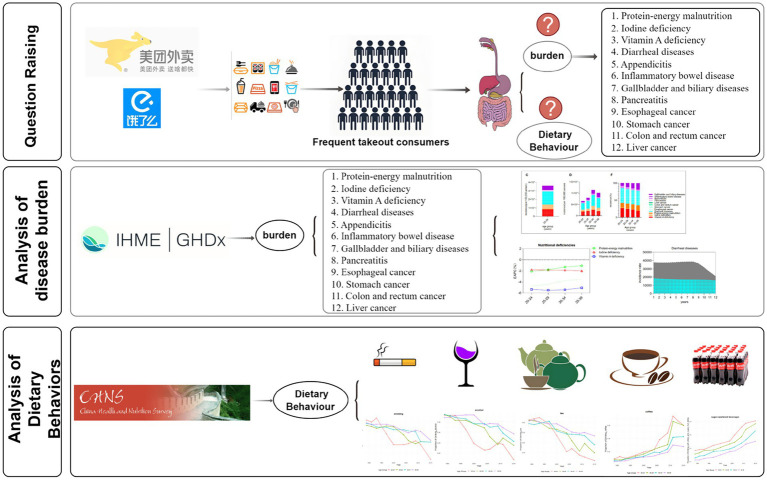

## Implications and contribution

- Despite a thirtyfold increase in China’s takeout market since 2011, the health impacts on frequent consumers—particularly concerning diet-related diseases (nutritional deficiencies, gastrointestinal disorders, cancers) and shifts in dietary behaviors—remain underexplored.
- By leveraging data from GBD 2021 and CHNS, this study uniquely correlates the rising burden of colorectal cancer and increasing consumption of coffee and soft drinks with dietary changes driven by takeout consumption.
- This provides essential evidence for policymakers to address health risks among China’s youth, a critical concern amidst the rapid expansion of the food industry.

## 1 Introduction

Over the past 11 years, China’s takeout market has expanded by more than thirtyfold since 2011 (1), predominantly driven by young adults—a demographic that constitutes the core consumer base of food delivery platforms due to their pursuit of convenience and digital engagement. College students and post-90s individuals in their first marriage have become the main force of takeout consumption (2, 3). The two predominant food delivery platforms, Meituan, established in 2010, and Ele.me, founded in 2009, collectively process nearly two million orders daily (4). The advent of COVID-19 and subsequent lockdown measures have increasingly normalized takeout food as a viable alternative to home-cooked meals (5). Coupled with the accelerated pace of life, young adults, driven by time constraints, convenience, and the lure of novel culinary experiences, are showing a growing reliance on takeout options. This shift has profoundly impacted their eating habits and nutritional profiles, posing significant challenges to public health and the societal framework in China (6). Despite these dramatic changes, the epidemiological landscape, particularly concerning diet-related diseases such as nutritional deficiencies and gastrointestinal disorders (both benign and malignant), and alterations in dietary behaviors over the past decade, remains inadequately explored.

The Global Burden of Disease (GBD) 2021 stands as the premier global cancer database, managed by the International Agency for Research on Cancer (IARC) Global Cancer Observatory and the Institute for Health Metrics and Evaluation (IHME). Concurrently, the China Health and Nutrition Survey (CHNS) is an ongoing, collaborative international cohort study overseen by the Carolina Population Center at the University of North Carolina at Chapel Hill and the Institute of Nutrition and Health, Chinese Center for Disease Control and Prevention. This project assesses the implications of China’s socio-economic transformations on the health and nutritional status of its populace. It evaluates the effects on nutritional and health behaviors and outcomes through shifts in community organization and projects, as well as alterations in economic, demographic, and social factors at family and individual levels.

Predominantly, the demographic that frequently utilizes takeout services in China includes college students and young professionals who are yet to establish families, generally aged between 20 and 40. These disease categories were selected due to their established or plausible links to dietary patterns commonly associated with frequent takeout consumption, which often features high energy density, low micronutrient content, and increased intake of processed foods, red meat, and sugary beverages—all of which are risk factors for nutritional deficiencies, gastrointestinal disorders, and certain cancers. This research, therefore, leverages data from the GBD 2021 and CHNS databases to investigate the current prevalence of twelve diet-related diseases among this young cohort (aged 20–39). The conditions studied include nutritional deficiency diseases, non-neoplastic gastrointestinal disorders, and gastrointestinal cancers, alongside shifts in dietary habits. The objective is to furnish detailed and reliable data that can inform governmental policy-making in the regulation of the takeout food sector.

## 2 Methods

### 2.1 Study overview

We employed the Global Burden of Disease 2021 (GBD 2021) database to ascertain the estimated number of cases [95% uncertainty interval (UI)] and standardized incidence rates (number of cases per 100,000 population) [95% confidence interval (CI)] for 12 diet-related diseases from 2010 to 2021. These diseases included nutritional deficiency disorders such as protein-energy deficiency, vitamin A deficiency, and iodine deficiency; non-neoplastic digestive system diseases including inflammatory bowel disease, pancreatitis, cholecystitis, appendicitis, and diarrheal diseases; and digestive system malignancies such as esophageal cancer, gastric cancer, liver cancer, and colorectal cancer. We also analyzed the temporal trends in disease burden comparing the periods 2010–2021 and 2000–2009, as well as the risk factor-attributable standardized disability-adjusted life years (DALYs). Additionally, we extracted data concerning dietary behaviors of the Chinese population within the target age group from the CHNS database to assess changes in dietary behaviors among young Chinese adults from 1990 to 2015.

### 2.2 Data origin

#### 2.2.1 GBD 2021

The GBD 2021 data^1^ encompass comprehensive estimates for 371 diseases and injuries and 88 risk factors across 204 countries and regions, and 811 sub-national regions spanning from 1990 to 2021. The dataset includes detailed metrics on incidence, mortality, prevalence, years lived with disability, DALYs, and healthy life expectancy, segmented by geographical location, year, age, gender, and socioeconomic indicators [Socio-Demographic Index (SDI)]. The GBD disease burden estimates incorporate diverse data sources, including population censuses, household surveys, civil registration and vital statistics, disease registries, health service utilization records, air pollution monitoring, satellite imagery, disease notifications, among others. This study conformed to the Guidelines for Accurate and Transparent Health Estimates Reporting (GATHER) and was exempt from review by the University of Toledo Institutional Review Board due to its use of publicly accessible data from the GHDx query tool.

#### 2.2.2 CHNS

Initiated in 1989, the CHNS captures extensive data on families, communities, and health institutions, including smoking status, and the consumption of tea, coffee, and alcohol, along with diet and activity levels for individuals aged 12 and older. Employing multi-stage random cluster sampling, the survey spans 15 provinces and municipalities, covering approximately 4,400 households and 19,000 individuals. Data collection is conducted through 7-day home visits that gather socio-economic and demographic data at both the household and individual levels, as well as community-level public resource information such as food markets and health facilities. Since 2011, the inclusion of three first-tier cities and three additional provinces in 2015—fully funded by Chinese partners—has broadened the survey’s scope. The 2015 dataset, which is publicly accessible, offers a longitudinal integrated file that facilitates the linkage and analysis of data across families and individuals.

### 2.3 Study population and rationale

As the GBD and CHNS lack direct measures of takeout consumption, we analyzed all adults aged 20-39—a demographic consistently shown to be the primary consumer base for food delivery platforms (2, 3). This group serves as a relevant proxy for evaluating ecological associations between takeout culture and health trends.

### 2.4 Definition

This study examines 12 diet-related diseases, each classified according to the disease codes from the 10th revision of the International Classification of Diseases and Related Health Problems (ICD-10). The diseases and their corresponding codes include: protein-energy malnutrition (E43-E46), vitamin A deficiency (E50), iodine deficiency (E00-E02), non-neoplastic digestive system diseases such as inflammatory bowel disease (K50-K51), acute pancreatitis (K85), cholecystitis (K81), appendicitis (K35-K38), non-infectious gastroenteritis and colitis (K52.9), and digestive system malignancies (esophageal cancer C15, gastric cancer C16, liver cancer C22, colon cancer C18, rectal cancer C19-C20, and anal cancer C21). It should be noted that colorectal cancer encompasses colon, rectum, and anus (C18-C21).

### 2.5 Incidence rate estimation

The GBD 2021 utilized standardized methodologies to derive disease estimates over various dimensions such as time, age, geography, health causes, and domains, integrating all accessible data. These estimates are presented with 95% CIs. The prior distributions in the Bayesian meta-regression tool, DisMod-MR, were adjusted based on simulation studies, which demonstrated that less informative priors enhanced the coverage of confidence intervals (7).

### 2.6 Temporal trend analysis

The Joinpoint regression model employs a series of linear statistical models, utilizing the least squares method to discern patterns in incidence rates, thereby minimizing subjectivity in trend analyses. Turning points are determined by minimizing the sum of squared residuals between the estimated and actual values. Natural logarithmic regression is applied to fit incidence rates for varying periods, and the annual percentage change (APC), along with its 95% CI, is computed for each period. In this research, the APC was calculated from the weighted average of slope coefficients derived from the joinpoint regression (8).

### 2.7 Risk factors

The risk factor estimation in the GBD adheres to a comparative risk assessment framework comprising six steps:

1. Identification of risk-outcome pairs that satisfy the World Cancer Research Fund criterion 18, supported by compelling or plausible evidence.
2. Quantification of the relationship between exposure and relative risk (RR) for each validated risk-outcome pair.
3. Distribution of exposure levels of each risk factor across different ages, genders, locations, and years.
4. Establishment of the theoretical minimum risk exposure level (TMREL).
5. Calculation of the population attributable fraction (PAF) and the attributable burden using RR, exposure levels, and TMREL. Multiplying PAF by DALYs determines the attributable DALYs for each risk factor.
6. Computation of the overall PAF and attributable burden for all identified risk factors.

DALYs, or Disability-Adjusted Life Years, represent a comprehensive metric of disease burden, amalgamating Years of Life Lost due to Premature Mortality (YLL) and Years Lived with Disability (YLD) to quantify the total loss of healthy life years. This study assessed the percentage contribution of level 3 risk factors to DALYs for specific diseases such as esophageal cancer, diarrheal diseases, colon and rectum cancer, and stomach cancer in the years 2010 and 2021. The selection of these four specific diseases for risk factor analysis was based solely on the availability of detailed, attributable burden data in the GBD 2021 database for the studied time period. Comparable data for the other diet-related diseases were not available.

### 2.8 Dietary behaviors

The analysis of dietary behaviors entails a structured process, as detailed below:

1. Retrieve data by accessing the CHNS data repository. Download the files titled “Master_ID_201908” and “Master_PE_PA_201908” located under the directory “Home/Survey Data/Data Sets/Data Downloads/Longitudinal.”
2. From the file “surveys_pub_12.sas7bdat” within “Master_ID_201908,” extract the variables “IDIND,” “AGE,” and “WAVE.”
3. From the file “pexam_pub_12.sas7bdat” within “Master_PE_PA_201908,” extract the variables “IDIND,” “U25” (Caloric Intake), “U34” (Year of Survey), “U37” (Smoking Status), “U40” (Tea Consumption), and “U229” (Consumption of Sugared Drinks).
4. Merge the extracted data using “IDIND” as a unique identifier and categorize individuals into newly defined age groups (20–24, 25–29, 30–34, 35–39) to create the variable “AgeGroup.”
5. Employ R to conduct a trend analysis of the variables from 1990 to 2015, focusing on changes within the defined age groups. The code used for this analysis is documented in Appendix S1.

### 2.9 Statistical analyses

We employed the estimated annual percentage change (EAPC) to evaluate the trend in the burden of twelve diet-related diseases from 2010 to 2021. A regression model, specifically a natural logarithmic model, was formulated as y = *α* + *β*x + ɛ, where y represents the natural logarithm of the age-standardized rate (ASR) and x denotes the calendar year. The EAPC of the ASR was derived using the formula 100 × (exp(β)−1), and the 95% CI was also calculated. Furthermore, we assessed the composition and proportion of age-standardized prevalence rates across four age groups (20–24, 25–29, 30–34, 35–39) using GraphPad Prism (version 8.0) and R (version 4.2.3).

## 3 Results

### 3.1 Main findings

#### 3.1.1 Incidence of 12 diet-related diseases among individuals aged 20–39 in China, 2010 and 2021

The data presented in Table 1, derived from the Global Burden of Disease Study 2021 (see Appendix S2), indicate the estimated number of cases and age-standardized incidence rates (ASIR) for 12 diet-related diseases in 2010 and 2021.

**Table 1:** The number of occurrences and standardized incidence rates of 12 diet-related diseases among Chinese young adults aged 20–39 in 2010 and 2021 from GBD 2021.

Disease	2010		2021	
	Incidence (No. cases) (95% UI)	ASIR per 100,000 people (95% CI)	Incidence (No. cases) (95% UI)	ASIR per 100,000 people (95% CI)
Nutritional deficiencies				
1 Protein-energy malnutrition	6,199,162.9 (8,157,170.5–4,682,539.5)	5,572.1 (7,326.1–4,214.3)	5,034,972.7 (6,299,315.5–4,040,569.1)	5,118.9 (6,418.9–4,095.4)
2 Iodine deficiency	710,409.0 (912,811.7–525,896.7)	626.6 (804.3–464.3)	457,505.4 (587,912.3–335,175.5)	513.4 (659.9–375.0)
3 Vitamin A deficiency	18,122,066.5 (29,091,397.4–10,722,790.3)	16,265.9 (2,107.1–9,615.7)	8,457,419.6 (14,041,323.9–4,755,489.0)	8,854.2 (14,693.8-4,977.8)
Non-neoplastic digestive system diseases				
1 Diarrheal diseases	20,430,303.4 (26,807,572.1–15,151,457.4)	18,344.1 (24,019.0–13,646.2)	15,181,986.7 (19,700,035.4–11,314,075.4)	15,694.4 (20,517.2–11,597.2)
2 Appendicitis	2,194,560.1 (3,488,289.7–1,283,283.4)	1,960.9 (3,123.1–1,146.6)	1,834,403.4 (2,946,825.5–1,064,497.5)	1,938.6 (3,097.6-1,122.0)
3 Inflammatory bowel disease	10,320.3 (13,578.9–7,798.4)	9.3 (12.3–7.1)	6,727.0 (9,154.05–4,924.4)	6.7 (9.1–4.9)
4 Gallbladder and biliary diseases	5,722,078.7 (7,491,378.0–4,248,711.2)	5,190.6 (6,796.5–3,857.3)	5,722,078.7 (7,491,378.0–4,248,711.2)	4,980.5 (6,546.9–3,695.4)
5 Pancreatitis	88,133.4 (134,262.0–53,426.4)	80.0 (122.0–48.4)	82,806.1 (125,618.3–49,857.4)	80.3 (121.6–48.6)
Gastrointestinal cancers				
1 Esophageal cancer	3,469.9 (3,915.3–3,065.1)	3.1 (3.5–2.7)	3,177.4 (3,946.3–2,551.7)	3.0 (3.7–2.4)
2 Stomach cancer	20,859.9 (24,135.8–16,921.7)	18.9 (21.8–15.3)	18,805.3 (23,183.7–14,652.8)	17.7 (21.8–13.8)
3 Colon and rectum cancer	20,315.9 (22,686.1–18,184.4)	18.4 (20.6–16.5)	27,952.1 (34,265.3–22,643.2)	26.4 (32.4–21.4)
4 Liver cancer	11,625.2 (13,139.4–10,296.3)	10.5 (11.8–9.3)	11,730.3 (15,223.1–9,045.6)	11.0 (14.3–8.5)

ASIR, age-standardized incidence rate; UI, uncertainty interval; CI, confidence interval.

Nutritional Deficiency Diseases: Over the eleven-year period, there was a notable decrease in ASIRs for these conditions. Vitamin A deficiency experienced the most substantial reduction, decreasing from 16,265.9 per 100,000 (95% UI: 26,107.1 to −9,615.7) in 2010 to 8,854.2 per 100,000 (95% UI: 14,693.8 to −4,977.8) in 2021.

Non-Neoplastic Digestive System Diseases: While the ASIR for Pancreatitis remained stable at approximately 80.0 per 100,000 (95% UI: 122.0 to 48.4) in 2010 and 80.3 per 100,000 (95% UI: 121.6 to 48.6) in 2021, the rates for other diseases in this category have declined. Diarrheal diseases showed the most significant decrease, from 18,344.1 per 100,000 (95% UI: 24,019.0 to 13,646.2) in 2010 to 15,694.4 per 100,000 (95% UI: 20,517.2 to 11,597.2) in 2021.

Gastrointestinal Cancers: The ASIRs for colon and rectum cancer and liver cancer have escalated. Specifically, colon and rectum cancer increased from 18.4 per 100,000 (95% UI: 20.6 to 16.5) in 2010 to 26.4 per 100,000 (95% UI: 32.4 to 21.4) in 2021, and liver cancer rose from 10.5 per 100,000 (95% UI: 11.8 to 9.3) in 2010 to 11.0 per 100,000 (95% UI: 14.3 to 8.5) in 2021.

#### 3.1.2 Proportional burden of twelve diet-related diseases in 2010 and 2021

The analysis, based on data from the Global Burden of Disease Study 2021 (refer to Appendix S3), delineates the proportional incidence rates alongside the absolute and relative burdens of twelve diet-related diseases affecting Chinese individuals aged between 20 and 39 during the years 2010 and 2021.

**Figure 1:**
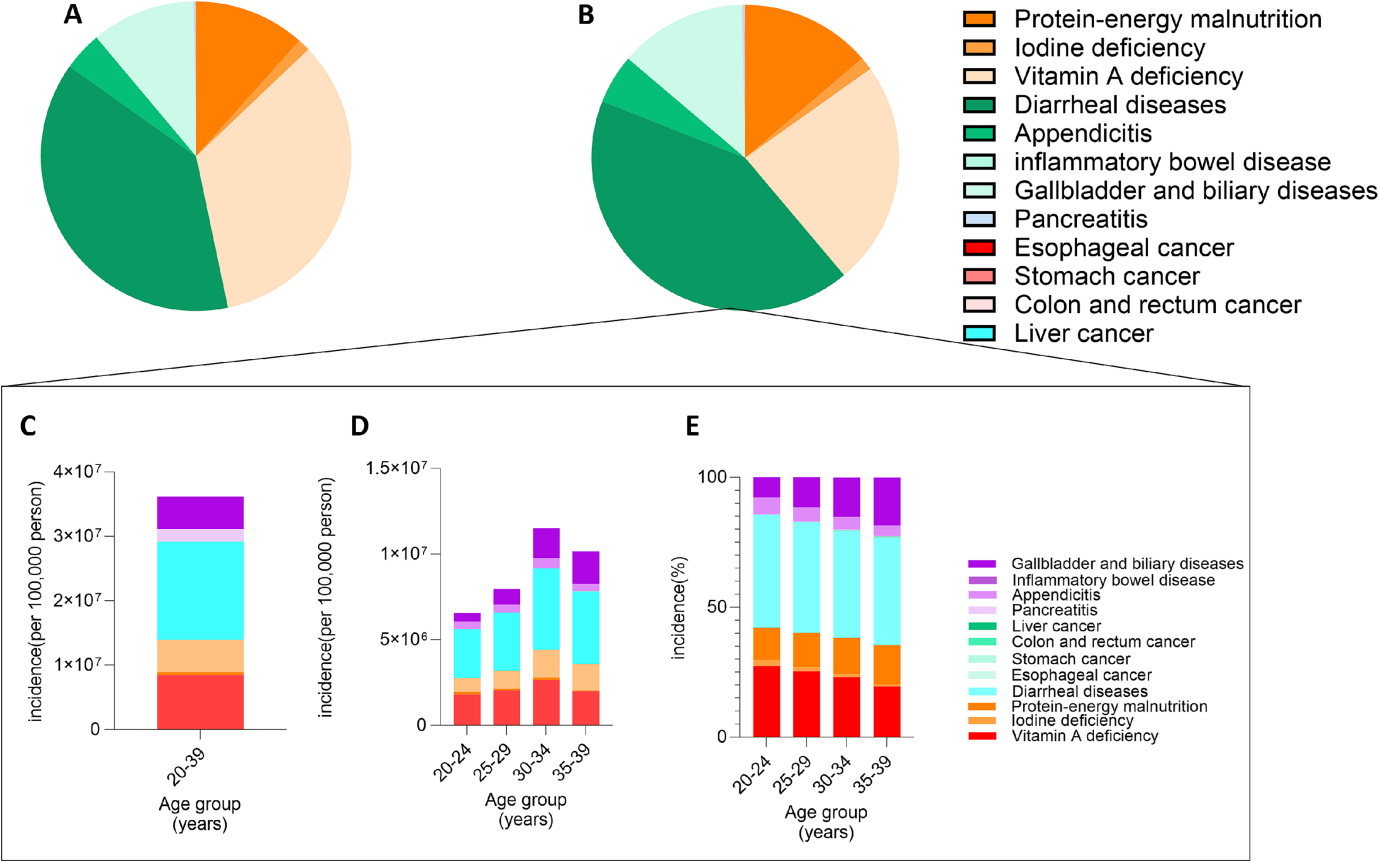
Variations in the proportional incidence of twelve diet-related diseases among Chinese Aged 20–39. **(A)** Standardized incidence rates of the diseases in 2010; **(B)** Standardized incidence rates of the diseases in 2021; **(C)** Absolute burden of the diseases in 2021; **(D)** Absolute burden segmented by five-year age groups in 2021; **(E)** Proportional burden segmented by five-year age groups in 2021.

Figures 1A,B illustrate that diarrheal diseases, vitamin A deficiency, and protein-energy malnutrition persistently emerged as the three most prevalent conditions by standardized incidence rate for both observed years. Figure 1C highlights that diarrheal diseases commanded the highest absolute burden, with vitamin A deficiency following. Figure 1D discloses that the aggregate disease burden peaked among individuals aged 30–34 and was least pronounced among those aged 20–24. Furthermore, Figure 1E illustrates a rising trend in the proportion of gallbladder and biliary diseases with advancing age, whereas the proportions of vitamin A and iodine deficiencies exhibited a decline.

#### 3.1.3 Estimated annual percentage change by five-year age group from 2010 to 2021

**Figure 2:**
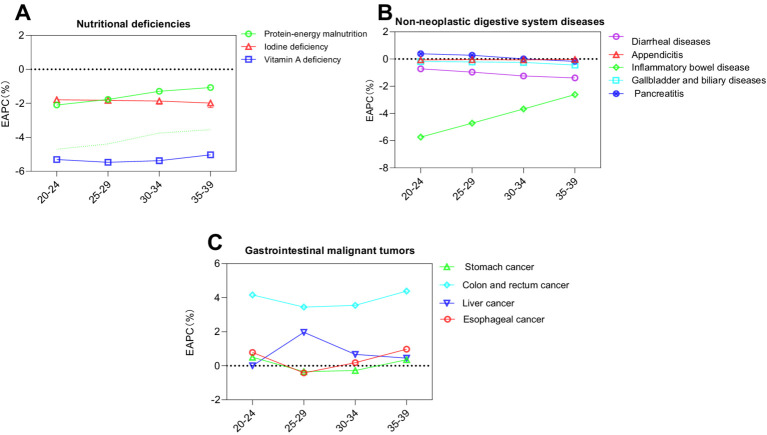
Estimated annual percentage change in the standardized incidence rates of twelve diet-related diseases across various age groups from 2010 to 2021 as presented in GBD 2021. **(A)** Nutritional deficiencies; **(B)** Non-neoplastic digestive system diseases; **(C)** Gastrointestinal cancers.

**Figure 3:**
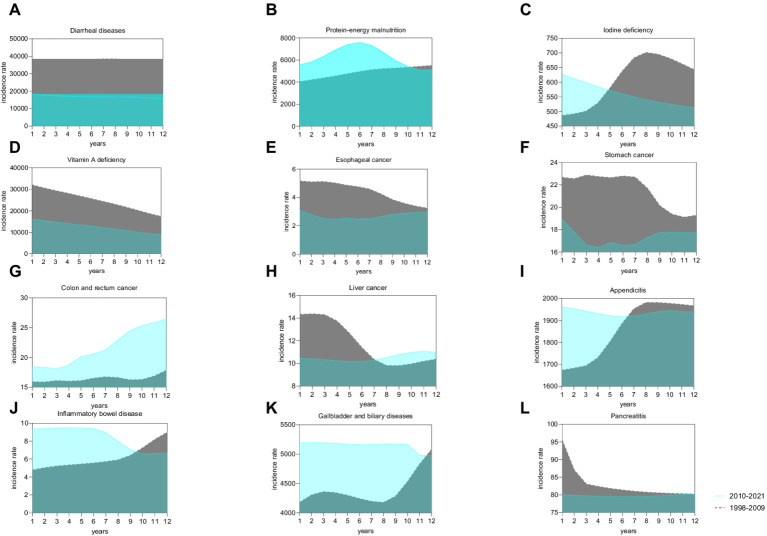
Comparative analysis of the incidence trends of twelve types of diet-related diseases among the Chinese population aged 20–39 during the intervals of 1998–2009 and 2010–2021. **(A)** Diarrheal diseases; **(B)** Protein-energy malnutrition; **(C)** Iodine deficiency; **(D)** Vitamin A deficiency; **(E)** Esophageal cancer; **(F)** Stomach cancer; **(G)** Colon and rectum cancer; **(H)** Liver cancer; **(I)** Appendicitis; **(J)** Inflammatory bowel disease; **(K)** Gallbladder and biliary diseases; **(L)** Pancreatitis.

The data extracted from Table 2 and Figures 2, 3, sourced from the Global Burden of Disease Study 2021 (refer to Appendix S4), outline the EAPC for these twelve diseases among Chinese individuals aged 20–39 across the period from 2010 to 2021.

**Table 2:** Estimated annual percentage change by five-year age group of twelve diet-related diseases from 2010 to 2021.

Disease	EAPC (%)			
	20–24	25–29	30–34	35–39
Nutritional deficiencies				
1 Protein-energy malnutrition	−2.10 (−4.70, 0.58)	−1.77 (−4.39, 0.92)	−1.29 (−3.75, 1.24)	−1.07 (−3.55, 1.47)
2 Iodine deficiency	−1.79 (−1.93, −1.65)	−1.82 (−1.95, −1.69)	−1.86 (−2.06, −1.66)	−1.98 (−2.26, −1.70)
3 Vitamin A deficiency	−5.31 (−5.66, −4.95)	−5.47 (−5.82, −5.12)	−5.38 (−5.73, −5.03)	−5.03 (−5.37, −4.69)
Non-neoplastic digestive system diseases				
1 Diarrheal diseases	−0.72 (−0.99, −0.44)	−0.96 (−1.23, −0.68)	−1.25 (−1.50, −1.00)	−1.39 (−1.61, −1.18)
2 Appendicitis	−0.06 (−0.21, 0.09)	−0.05 (−0.17, 0.07)	−0.06 (−0.16, 0.04)	−0.03 (−0.16, 0.11)
3 Inflammatory bowel disease	−5.74 (−7.13, −4.33)	−4.71 (−6.05, −3.35)	−3.67 (−5.03, −2.28)	−2.61 (−3.86, −1.35)
4 Gallbladder and biliary diseases	−0.19 (−0.42, 0.04)	−0.21 (−0.45, 0.03)	−0.25 (−0.48, −0.03)	−0.45 (−0.63, −0.28)
5 Pancreatitis	0.39 (0.34, 0.43)	0.28 (0.20, 0.37)	0.01 (−0.08, 0.09)	−0.17 (−0.21, −0.14)
Gastrointestinal cancers				
1 Esophageal cancer	0.78 (−0.23, 1.81)	−0.42 (−1.31,0.48)	0.18 (−1.32, 1.70)	0.98 (−0.72, 2.71)
2 Stomach cancer	0.50 (−0.21, 1.22)	−0.34 (−0.65, −0.03)	−0.28 (−1.15, 0.60)	0.36 (−0.62, 1.35)
3 Colon and rectum cancer	4.16 (3.55, 4.77)	3.45 (3.07, 3.83)	3.55 (2.89, 4.21)	4.39 (3.62, 5.16)
4 Liver cancer	0.00 (−2.16, 2.20)	1.97 (1.12, 2.82)	0.67 (0.59, 0.74)	0.45 (−0.29, 1.21)

Regarding nutritional deficiencies, conditions such as protein-energy malnutrition, iodine deficiency, and vitamin A deficiency all registered a declining trend, each with an EAPC less than zero.

In the category of non-neoplastic digestive system diseases, pancreatitis demonstrated an upward trend (EAPC > 0) in the age cohorts of 20–24, 25–29, and 30–34. Conversely, inflammatory bowel disease exhibited an age-associated decrease, more pronounced in younger groups, with the absolute value of EAPC increasing as age decreased.

Among gastrointestinal cancers, both colon and rectal cancer, as well as liver cancer, displayed upward trends (EAPC > 0) across all age groups. Esophageal cancer showed a decreasing trend [EAPC = −0.42 (−1.31, 0.48)] in the 25–29 age group. Stomach cancer exhibited upward trends in the age groups of 20–24 and 35–39 [EAPC = 0.50 (−0.21, 1.22) and 0.36 (−0.62, 1.35), respectively].

#### 3.1.4 Temporal trends

Figure 3 provides a comparative analysis of the standardized incidence rates for twelve diet-related diseases between two periods: 1998–2009 and 2010–2021, with the original data detailed in Appendix S5. Notably, the incidence rate of colorectal cancer was consistently higher during the latter period (2010–2021) compared to the earlier period (1998–2009). In contrast, the incidence rates for several other conditions, including diarrheal diseases, vitamin A deficiency, esophageal cancer, stomach cancer, and pancreatitis, showed a decline during 2010–2021.

#### 3.1.5 DALYs attributable to risk factors (2010–2021)

**Figure 4:**
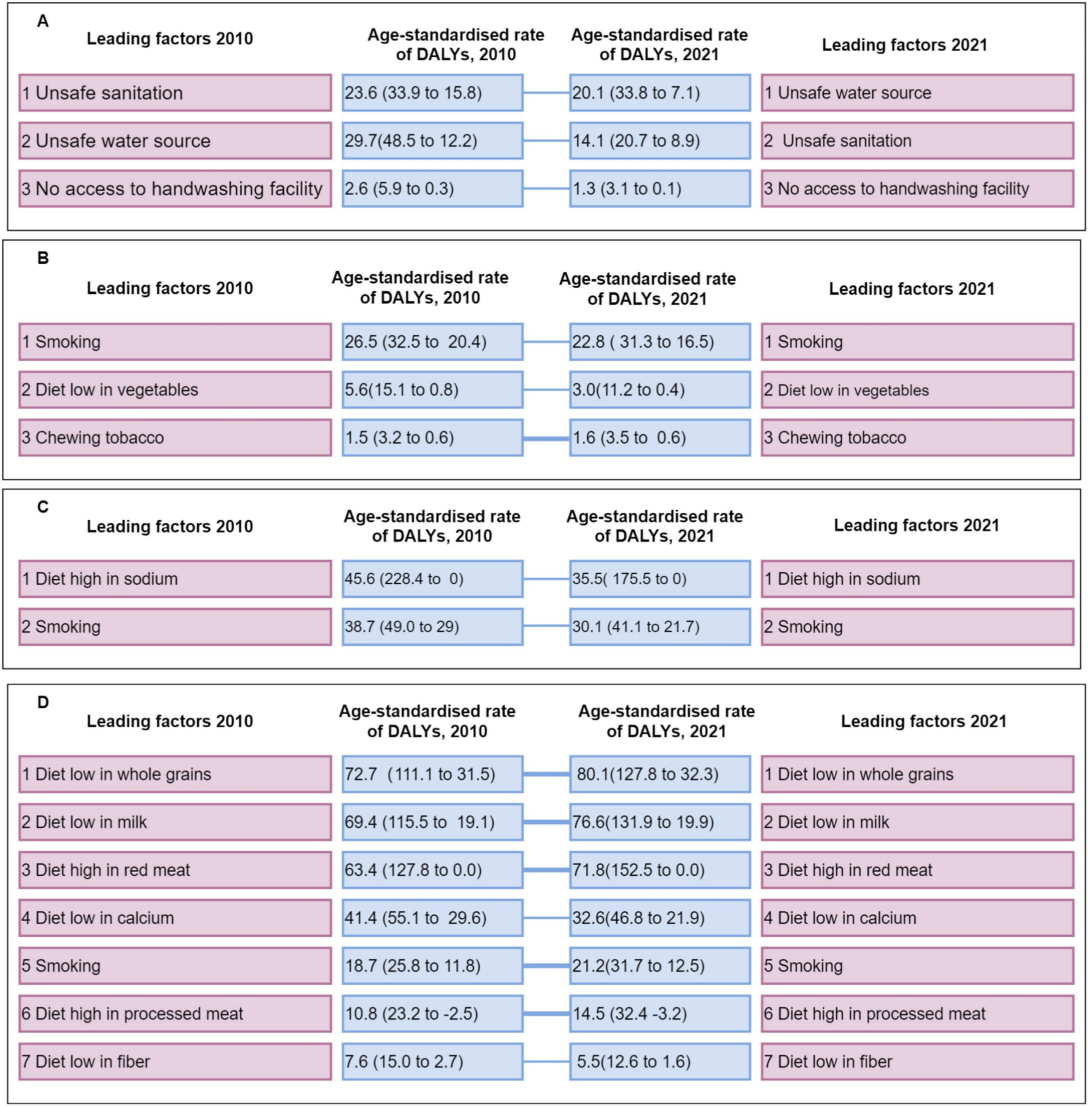
All level 3 risk factors for age-standardized rates of DALYs and the movement of these risk factors into or out of the top ten between 2010 and 2021 for the Chinese population aged 20–39, covering both sexes. **(A)** Diarrheal diseases; **(B)** Esophageal cancer; **(C)** Stomach cancer; **(D)** Colon and rectum cancer. The graphical representation uses thin blue lines to indicate a decrease in ranking and thick blue lines to denote an increase in DALYs, with the data enclosed in parentheses representing the 95% UI. The level 3 risk factors are part of the Global Burden of Disease Study’s classification system, which ranges from level 2 to level 4. DALYs denote disability-adjusted life-years.

Figure 4, derived from the Global Burden of Disease Study 2021 (refer to Appendix S6), illustrates the variations in DALYs attributed to level 3 risk factors for four specific diseases between 2010 and 2021.

In the case of colorectal cancer, significant increases were observed in DALYs associated with diets low in whole grains [from 72.7 (111.1 to 31.5) to 80.1 (127.8 to 32.3)], high in red meat [from 63.4 (127.8 to 0.0) to 71.8 (152.5 to 0.0)], and smoking [from 18.7 (25.8 to 11.8) to 21.2 (31.7 to 12.5)]. Additionally, DALYs related to a high intake of processed meat increased from 10.8 [23.2 to −2.5] to 14.5 [32.4 to −3.2].

For the other three diseases analyzed, with the exception of an increase in DALYs from chewing tobacco associated with esophageal cancer, the rankings of the level 3 risk factors remained largely unchanged, and the overall DALYs values generally declined.

The graphical representation uses thin blue lines to indicate a decrease in ranking and thick blue lines to denote an increase in DALYs, with the data enclosed in parentheses representing the 95% UI. The level 3 risk factors are part of the Global Burden of Disease Study’s classification system, which ranges from level 2 to level 4. DALYs denote disability-adjusted life-years.

#### 3.1.6 Alterations in dietary behaviors

**Figure 5:**
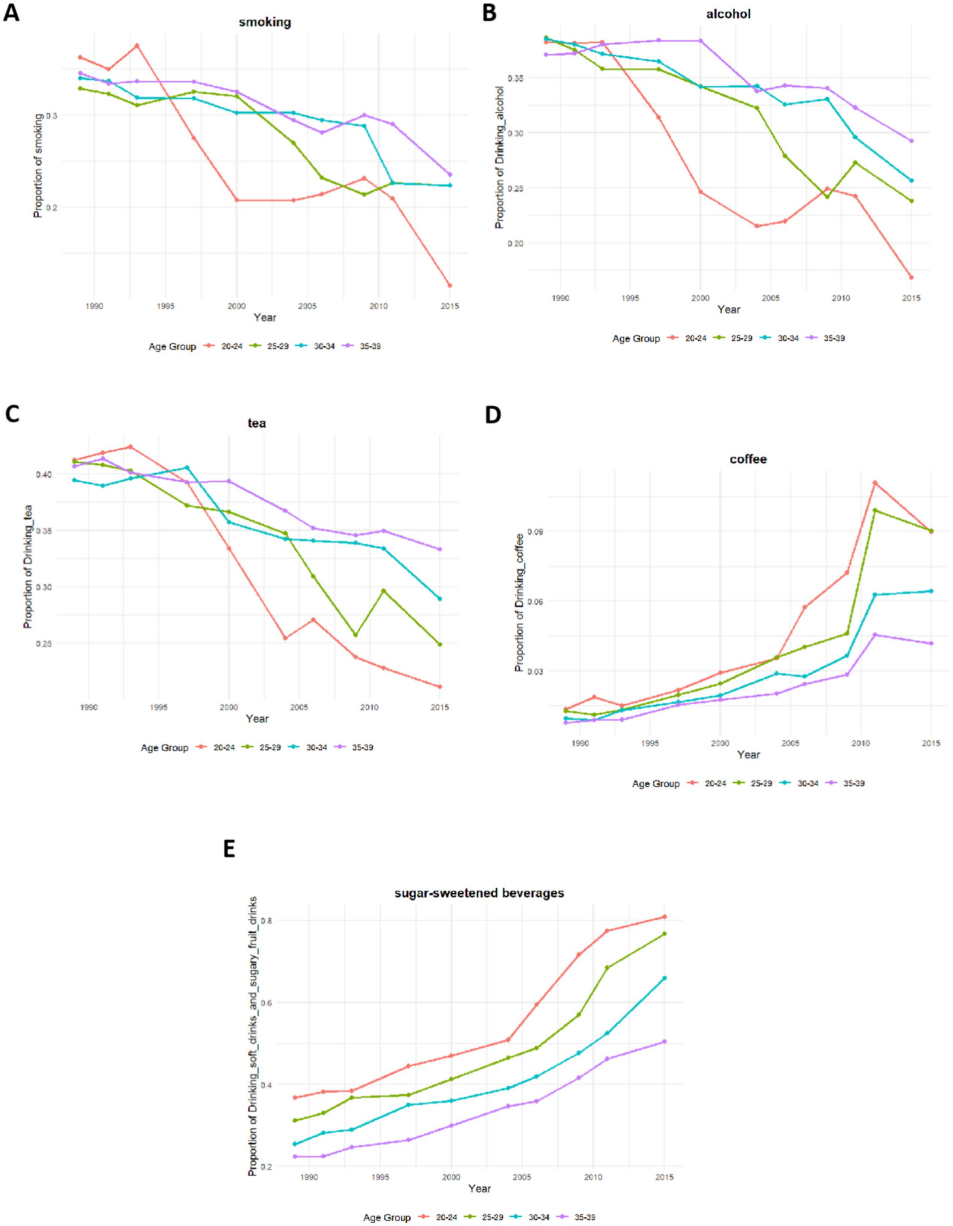
Trends in dietary behavior changes among individuals aged 20–39, 1990–2015. **(A)** Smoking; **(B)** Alcohol consumption; **(C)** Tea consumption; **(D)** Coffee consumption; **(E)** Soft drink and sugary beverage consumption.

Data pertaining to behavioral habits were extracted from the CHNS database, spanning from 1990 to 2015, encompassing longitudinal observations of 199,281 individuals. The dataset includes variables such as smoking, tea and coffee consumption, alcohol use, and the intake of soft drinks and sugary beverages. As depicted in Figures 5A–C, there was a notable decline in the percentages of individuals aged 20–39 who smoked, consumed alcohol, or drank tea over the 25-year period. Conversely, Figures 5D,E illustrate that the consumption of coffee and soft drinks progressively increased across all age groups during these years. It is important to note that after 2010, the proportion of coffee consumers in the 25–29 and 30–34 age brackets showed a decline.

## 4 Discussion

This study elucidates several trends within the target demographic. Notably, there has been a consistent, linear reduction in Vitamin A deficiency over the past 11 years. Among non-neoplastic gastrointestinal diseases, IBD exhibited the most significant annual decrease in standardized incidence rates. In contrast, the disease burden associated with colorectal cancer has escalated annually across all age groups. Moreover, DALY rates attributed to various risk factors for colon and rectum cancer—including insufficient whole grain and milk intake, along with excessive consumption of red meat, smoked products, and tobacco—have increased in 2021 relative to 2010. Concurrently, dietary shifts around 2010 were marked by a turnaround in coffee consumption and persistently rising soft drink intake, coinciding with the rapid expansion of China’s takeout industry.

The study also highlights a decrease in the incidences of three types of nutritional deficiencies among young adults in China. While protein-energy malnutrition and dietary iron deficiency predominantly affect those over 70 and under 5 years of age, regional disparities exist. In provinces such as Zhejiang, Beijing, and Guangdong, the prevalent forms of malnutrition are protein-energy malnutrition and Vitamin A deficiency, whereas in Tibet, Xinjiang, and Hainan, dietary iron deficiency and protein-energy malnutrition are more common (9). This comprehensive study encompasses all age groups and offers regional insights into nutritional deficiencies in China, thereby augmenting the GBD 2021 data, which lacks province-specific disease burden information. The notable reduction in malnutrition incidences can be attributed to rapid economic growth and recent policy implementations, such as the “Healthy China 2030 Plan Outline” and the “Healthy China Initiative (2019–2030),” which have focused on promoting nutrition and health, thereby improving the nutritional status of young adults (10). Since the adoption of the “Iodized Salt Law” in 1994, iodine deficiency-related health issues, including cognitive impairments, growth disorders, and thyroid diseases, have been effectively mitigated (11). An appropriate daily intake of iodine from iodized salt (150–250 μg for adults) is crucial for minimizing thyroid dysfunction (12). A comprehensive study involving 78,470 adults across 31 provinces demonstrated a 95.37% coverage rate of iodized salt and a goiter prevalence of 1.17% (95% CI: 0.95–1.43%) (13). Further research indicates that young men may achieve their minimal iodine requirements with approximately half the currently recommended daily intake (120 μg/day) (14).

The reduction in vitamin A deficiency observed from 2010 to 2021 was notably significant among the three types of malnutrition disorders examined. Vitamin A, a crucial fat-soluble micronutrient, plays an essential role in preventing vision loss (15) and dry eye syndrome (16). Vegetables are the primary contributors to daily vitamin A intake, accounting for 54.94% (253.03 μg RAE/day), followed by eggs, milk, aquatic products, meat, fruits, beans, coarse grains, and potatoes (17). An analysis of cross-sectional data from 12,246 Chinese adults aged 18–64 in 2015 indicated that approximately 87% of the population consumed less vitamin A than the Estimated Average Requirement (EAR), and only 6% met or exceeded the Recommended Nutrient Intake (RNI) (18). Although this study suggests a substantial reduction in vitamin A deficiency among young adults, overall adult intake may still be inadequate. This underscores the urgency of further delineating epidemiological characteristics to aid governments and organizations in developing more targeted and effective health policies.

The annual standardized incidence rate of IBD in the 20–39 age group within non-neoplastic digestive system diseases has shown the most pronounced decline. This apparent contradiction underscores the significant geographical and demographic heterogeneity in IBD trends globally (19–21). For instance, while China has experienced a rapid rise in ASIR (EAPC = 2.93), a slight but significant global increase has been observed in the 10–24 age group, even as the 50–69 age group shows a decline (19, 21). Identified risk factors include smoking and appendectomy (22), while an imbalance in intestinal flora is also considered a critical factor (23). Several non-mutually exclusive factors may explain the declining trend observed in our data: (1) genuine improvements in food safety, hygiene, and environmental factors in certain regions may have reduced microbial triggers; (2) increased health awareness and dietary modifications may be mitigating risk or delaying progression; (3) methodological differences, including the use of aggregated GBD estimates versus hospital-based or regional cohort studies, can capture varying aspects of the epidemiological landscape. Therefore, the decline reported here does not negate the rising burden elsewhere but highlights the shifting and complex nature of IBD epidemiology, influenced by local genetic, environmental, and healthcare factors.

This study demonstrates that the incidence of colorectal cancer among individuals aged 20–39 is increasing annually. Early-onset cancer (EOC) generally refers to cases diagnosed in patients under the age of 50 (24). Over the last three decades, the ASIR, mortality rate, and DALYs associated with colorectal cancer have shown the most rapid increase, with an AAPC of 3.06 (*p* < 0.001) (25). The dataset comprised 8,465 patients diagnosed with primary colorectal cancer across 13 tertiary hospitals in nine Chinese provinces from 2005 to 2014, with a mean age of 59.3 ± 12.8 years (26). Furthermore, a review of inpatient records from 2014 to 2018 at hospitals affiliated with Peking University included 2,097,347 cases; the rectum was identified as the most frequent site of cancer (48.3%), followed by the distal colon (24.5%) and the proximal colon (18.6%) (27). Patients diagnosed with early-stage colorectal cancer exhibited more aggressive disease, more advanced TNM staging, and a higher frequency of surgical and perioperative treatments (28). However, a 2019 study in Shenzhen, China, reported that only 15.6% of the population participated in colonoscopy screenings (29). This low screening rate suggests that the actual prevalence of early-onset colorectal cancer among young adults may exceed estimates from the Global Burden of Disease Study 2021 and underscores the importance of earlier screening, particularly for high-risk groups such as those with a familial history of colorectal cancer, prior polyps, or gastrointestinal symptoms.

The development of colorectal cancer results from interactions between genetic predispositions and environmental influences. Genetic factors determine an individual’s cancer susceptibility (30), while environmental and lifestyle factors such as physical inactivity, obesity, high alcohol intake, and smoking contribute to risk (31). In contrast to the increases observed in DALYs for several risk factors of colorectal cancer, the overall DALYs values for the other three diseases analyzed (diarrheal diseases, esophageal cancer, and stomach cancer) generally declined between 2010 and 2021, and the rankings of their level 3 risk factors remained largely unchanged (Figure 4). This highlights that the rising burden attributable to dietary and lifestyle risks is a particular concern for colorectal cancer among young adults in China. The prevalence of a diet high in processed sugars and red meat, referred to as the “sweet meat diet,” is considered a pathogenic factor within the Chinese population, particularly among those under 40 (32). A comparative study involving 222 patients with early-onset colorectal cancer (EOCRC) aged 30–50 and 87,833 controls found that a higher body mass index, frequent alcohol consumption, increased fish consumption, hypertension, diabetes, and having a first-degree relative with cancer were associated with an elevated risk of developing EOCRC (33). Smoking has also been identified as a significant risk factor (34). In addition to these lifestyle and clinical factors, broader macro-level determinants such as socioeconomic status (SES), urbanization, and healthcare access may also confound the observed trends. For instance, lower SES is associated with disparities in access to advanced cancer treatments and screening (35), and higher household income is linked to improved survival in colorectal cancer patients (36). However, further research is necessary to establish the causal relationships between these risk factors and EOCRC, and to enhance the effectiveness of targeted prevention strategies. A critical component of such strategies involves the expansion of colonoscopy screening programs targeted at young adults.

Furthermore, analysis of CHNS data revealed a marked transition in dietary behaviors around 2010. The decline in traditional practices (smoking, alcohol, and tea consumption) accelerated, while coffee consumption trend reversed from growth to decline—a shift potentially attributable to the substitution effect of emerging ready-to-drink tea beverages. In contrast, soft drink consumption demonstrated sustained and substantial growth. This rise may be facilitated by the proliferation of ‘sugar-free’ options alleviating health concerns, and importantly, was closely concurrent with the rapid expansion of the online food delivery industry, whose marketing strategies and convenience likely catalyzed soft drink consumption. Two studies utilizing the CHNS further enriched our findings, confirming that this trend reflects a shift in dietary preferences in China from traditional patterns toward more Western-influenced diets. An analysis of the CHNS encompassing 29,238 adults aged 18 and older, who had complete demographic and dietary records, revealed three distinct dietary patterns: the Southern pattern, characterized by a high intake of rice, vegetables, and pork, which showed a decrease in its score from 0.11 ± 1.13 in 1991 to −0.22 ± 0.93 in 2015; the Modern pattern, marked by increased consumption of fruits, dairy products, cakes, cookies, and pastries, which saw its score rise from −0.44 ± 0.59 in 1991 to 0.21 ± 1.01 in 2015; and the Meat pattern, which includes a higher intake of offal, poultry, and other livestock meats, and which also increased, from −0.18 ± 0.98 in 1991 to 0.27 ± 0.91 in 2015 (37). Further analysis by Bu (38) of data from the CHNS between 1997 and 2011 confirmed significant increases in the consumption of fruits, dairy products, snacks, fast food, and beverages, alongside a notable decrease in rice consumption.

Taken together, the evolving patterns of takeaway food consumption, shifts in dietary structure, and the rising incidence of gut-related diseases such as colorectal cancer suggest an ecological association worth attention. Although current evidence is insufficient to establish a causal relationship, these observations underscore the need for further investigation. Future research should consider integrating multiple data sources—such as online food delivery records, clinical health outcome databases, and national dietary surveys—to conduct large-scale, longitudinal studies. Such efforts would help elucidate the impact of the modern dietary environment on the chronic disease burden and provide critical evidence for public health policy and intervention strategies.

The primary limitations of this study include: First, as an ecological analysis, it relies on macro-level data from the GBD and CHNS, and we lacked direct individual-level data on takeout consumption frequency. Consequently, the proposed link with takeout culture is inferred from parallel population-level trends and does not establish direct causality. Second, the GBD framework does not provide subnational estimates, preventing us from exploring potential provincial-level variations in disease burden that might correlate with regional economic or dietary differences. Third, our assessment of risk factors was limited to the factors and disease pairs available in GBD 2021, which may not encompass the full spectrum of diet-related pathologies or emerging risk factors. Finally, the CHNS dietary behavior data, which ended in 2015, is outdated and may not fully capture the most recent and rapid shifts in food consumption patterns driven by the peak expansion of mobile food delivery platforms. Future studies integrating individual takeout records (e.g., from platform ordering data), more recent dietary surveys, and clinical health outcomes are essential to validate these ecological associations.

## 5 Conclusion

In conclusion, our ecological analysis documents parallel trends: a rising burden of colorectal cancer alongside a rapid shift in dietary behaviors and the expansion of the takeout market among young adults in China from 2010 to 2021. While these trends are suggestive, the evidence remains associative and does not establish causality. The findings highlight an urgent public health concern and generate a compelling hypothesis that frequent takeout consumption may be a contributing factor to the rising incidence of early-onset colorectal cancer. Future research must move beyond ecological correlations to investigate causal links, leveraging direct measures of takeout consumption from platform data and contemporary dietary surveys. Public health initiatives should simultaneously focus on promoting healthier dietary choices through these ubiquitous food delivery platforms.

## Implications and contribution

Despite a thirtyfold increase in China’s takeout market since 2011, the health impacts on frequent consumers—particularly concerning diet-related diseases (nutritional deficiencies, gastrointestinal disorders, cancers) and shifts in dietary behaviors—remain underexplored. By leveraging data from GBD 2021 and CHNS, this study uniquely correlates the rising burden of colorectal cancer and increasing consumption of coffee and soft drinks with dietary changes driven by takeout consumption. This provides essential evidence for policymakers to address health risks among China’s youth, a critical concern amidst the rapid expansion of the food industry.

## Data Availability

The original contributions presented in the study are included in the article/Supplementary material, further inquiries can be directed to the corresponding authors.

## Footnotes

^1^ http://ghdx.healthdata.org/gbd-2021/data-input-sources
