# Supplementary material for: Is takeout culture associated with rising colorectal cancer in young Chinese adults? An ecological analysis of GBD and CHNS data
Source: Front Public Health. 2025 Oct 23;13:1665377. doi: 10.3389/fpubh.2025.1665377 (PMC12589040; doi:10.3389/fpubh.2025.1665377)
Supplement: Supplementary file 1 [file Table_1.DOCX]

library(dplyr)

library(ggplot2)

library(haven)

setwd("D:/Rdata/CHNS/Master_ID_201908")

data_diet<- read_sas("surveys_pub_12.sas7bdat")

head(data_diet)

str(data_diet)

filtered_data_diet <- filter("data_diet, AGE >= 20 & AGE <= 39)

print(filtered_data_diet)

View(filtered_data_diet)

setwd("D:/Rdata/CHNS/Master_PE_PA_201908")

data_diet2<- read_sas("pexam_pub_12.sas7bdat")

head(data_diet2)

str(data_diet2)

selected_data_diet2 <- select(data_diet2, IDind,U25,U34,U35,U37,U40,U41,U229,U230)

print(selected_data_diet2)

setwd("D:/Rdata/CHNS/Master_ID_201908")

data_diet1<- read_sas("surveys_pub_12.sas7bdat")

head(data_diet)

str(data_diet)

selected_data_diet <- select(data_diet, Idind, age, wave)

new_selected_data_diet <- selected_data_diet%>%

rename(IDind = Idind)

print(new_selected_data_diet)

install.packages("data.table")

library(data.table)

diet1 <- as.data.table(new_selected_data_diet)

diet2 <- as.data.table(selected_data_diet2)

merged_data <- merge(new_selected_data_diet, selected_data_diet2, by = "IDind", all = TRUE) # all = TRUE

print(merged_data)

filtered_merged_data <- merged_data %>% filter(age >= 20 & age <= 39)

print(filtered_merged_data)

options(utils.rstudio.tools.pkgdown = TRUE)

filtered_merged_data_group <- filtered_merged_data %>%

mutate(AgeGroup = case_when(

age >= 20 & age <= 24 ~ "20-24",

age >= 25 & age <= 29 ~ "25-29",

age >= 30 & age <= 34 ~ "30-34",

age >= 35 & age <= 39 ~ "35-39",

TRUE ~ NA_character_

))

print(filtered_merged_data_group)

#Variable renaming

library(dplyr)

new_names <- c(

year = "wave", # 将 wave 改名为 year

smoking = "U25",

Drinking_tea = "U34",

Drinking_coffee = "U37",

Drinking_alcohol = "U40",

Drinking_soft_drinks_and_sugary_fruit_drinks = "U229"

)

print(names(filtered_merged_data_group))

filtered_merged_data_group <- filtered_merged_data_group %>%

rename(!!!new_names)

print(names(filtered_merged_data_group)

#smoking

test_data <- filtered_merged_data_group %>%

filter(!is.na(smoking)) %>% # 过滤掉 NA 值

group_by(year, AgeGroup, smoking) %>%

summarise(Count = n(), .groups = 'drop') %>% # 计算每个组的数量

group_by(year, AgeGroup) %>%

mutate(Proportion = Count / sum(Count)) %>% # 计算比例

filter(smoking == 1) # 只保留 smoking == 1 的数据

print(test_data)

test_plot <- ggplot(test_data, aes(x = year, y = Proportion, color = AgeGroup)) +

geom_line(size = 1) +

geom_point(size = 2) +

labs(

title = "smoking",

x = "Year",

y = "Proportion of smoking",

color = "Age Group"

) +

theme_minimal() +

theme(

plot.title = element_text(hjust = 0.5, size = 14, face = "bold"),

axis.title = element_text(size = 12),

legend.position = "bottom"

)

print(test_plot)

#Tea

test_data <- filtered_merged_data_group %>%

filter(!is.na(Drinking_tea)) %>% # 过滤掉 NA 值

group_by(year, AgeGroup, Drinking_tea) %>%

summarise(Count = n(), .groups = 'drop') %>% # 计算每个组的数量

group_by(year, AgeGroup) %>%

mutate(Proportion = Count / sum(Count)) %>% # 计算比例

filter(Drinking_tea == 1) # 只保留 smoking == 1 的数据

print(test_data)

test_plot <- ggplot(test_data, aes(x = year, y = Proportion, color = AgeGroup)) +

geom_line(size = 1) +

geom_point(size = 2) +

labs(

title = "tea",

x = "Year",

y = "Proportion of Drinking_tea",

color = "Age Group"

) +

theme_minimal() +

theme(

plot.title = element_text(hjust = 0.5, size = 14, face = "bold"),

axis.title = element_text(size = 12),

legend.position = "bottom"

)

print(test_plot)

#coffee

test_data <- filtered_merged_data_group %>%

filter(!is.na(Drinking_coffee)) %>% # 过滤掉 NA 值

group_by(year, AgeGroup, Drinking_coffee) %>%

summarise(Count = n(), .groups = 'drop') %>% # 计算每个组的数量

group_by(year, AgeGroup) %>%

mutate(Proportion = Count / sum(Count)) %>% # 计算比例

filter(Drinking_coffee == 1) # 只保留 smoking == 1 的数据

print(test_data)

test_plot <- ggplot(test_data, aes(x = year, y = Proportion, color = AgeGroup)) +

geom_line(size = 1) +

geom_point(size = 2) +

labs(

title = "coffee",

x = "Year",

y = "Proportion of Drinking_coffee",

color = "Age Group"

) +

theme_minimal() +

theme(

plot.title = element_text(hjust = 0.5, size = 14, face = "bold"),

axis.title = element_text(size = 12),

legend.position = "bottom"

)

print(test_plot)

#重复做图 alcohol

test_data <- filtered_merged_data_group %>%

filter(!is.na(Drinking_alcohol)) %>% # 过滤掉 NA 值

group_by(year, AgeGroup, Drinking_alcohol) %>%

summarise(Count = n(), .groups = 'drop') %>% # 计算每个组的数量

group_by(year, AgeGroup) %>%

mutate(Proportion = Count / sum(Count)) %>% # 计算比例

filter(Drinking_alcohol== 1) # 只保留 smoking == 1 的数据

print(test_data)

test_plot <- ggplot(test_data, aes(x = year, y = Proportion, color = AgeGroup)) +

geom_line(size = 1) +

geom_point(size = 2) +

labs(

title = "alcohol",

x = "Year",

y = "Proportion of Drinking_alcohol",

color = "Age Group"

) +

theme_minimal() +

theme(

plot.title = element_text(hjust = 0.5, size = 14, face = "bold"),

axis.title = element_text(size = 12),

legend.position = "bottom"

)

print(test_plot)

#重复做图 soft_drinks_and_sugary_fruit_drinks

test_data <- filtered_merged_data_group %>%

filter(!is.na(Drinking_soft_drinks_and_sugary_fruit_drinks)) %>% # 过滤掉 NA 值

group_by(year, AgeGroup, Drinking_soft_drinks_and_sugary_fruit_drinks) %>%

summarise(Count = n(), .groups = 'drop') %>% # 计算每个组的数量

group_by(year, AgeGroup) %>%

mutate(Proportion = Count / sum(Count)) %>% # 计算比例

filter(Drinking_soft_drinks_and_sugary_fruit_drinks== 1) # 只保留 smoking == 1 的数据

print(test_data)

test_plot <- ggplot(test_data, aes(x = year, y = Proportion, color = AgeGroup)) +

geom_line(size = 1) +

geom_point(size = 2) +

labs(

title = "sugar-sweetened beverages",

x = "Year",

y = "Proportion of Drinking_soft_drinks_and_sugary_fruit_drinks",

color = "Age Group"

) +

theme_minimal() +

theme(

plot.title = element_text(hjust = 0.5, size = 14, face = "bold"),

axis.title = element_text(size = 12),

legend.position = "bottom"

)

print(test_plot)
